# Nutrient Artery Canal of Femur Masquerading a Fracture Line: A Case Report

**DOI:** 10.7759/cureus.84306

**Published:** 2025-05-17

**Authors:** Anmol Anand, Nitish J Jyoti, Siva Srivastava Garika, Vijay Sharma, Shivanand Gamanagatti

**Affiliations:** 1 Orthopedics, All India Institute of Medical Sciences, New Delhi, New Delhi, IND; 2 Radiology, All India Institute of Medical Sciences, New Delhi, New Delhi, IND

**Keywords:** femur, fracture mimic, intertrochanteric fracture, nutrient artery canal, trochanteric femoral nail

## Abstract

Radiographic differentiation between true fracture lines and anatomical structures, such as nutrient artery canals, is crucial for optimal fracture management and postoperative mobilization. We present the case of a 54-year-old male with an AO 31A1.3 (AO/OTA 2018) pertrochanteric femur fracture treated with short trochanteric femoral nailing. Postoperative radiographs revealed an oblique radiolucent line adjacent to the distal part of the nail, raising suspicion of an undisplaced distal fracture extension. However, contralateral femoral imaging and CT scan confirmed the finding to be a nutrient artery canal. The patient was safely mobilized with full weight-bearing without the need for implant revision. Awareness of the radiographic features of nutrient artery canals is essential to avoid misinterpretation, unnecessary surgical modifications, and unwarranted delays in rehabilitation following fracture fixation.

## Introduction

Intramedullary devices offer superior stability in intertrochanteric fractures, facilitating early ambulation and accelerated functional recovery [1]. However, certain situations warrant caution with early weight bearing, such as cases with negative medial cortical reduction, severe postero-medial comminution, and multi-fragmentary intertrochanteric fractures [2]. Another potential concern is an undisplaced fine extension of the fracture line distally, which may go unnoticed, especially when planning for a short trochanteric femoral nail (TFN) [3]. Therefore, it is crucial to obtain comprehensive imaging of the entire femur.

While emphasizing the importance of thorough evaluation, it is equally critical to recognize normal radiographic entities that may mimic a fracture line, potentially leading to misinterpretation and impacting both surgical fixation strategy and postoperative mobilization protocols. One such entity is the nutrient artery canal, which can appear as an oblique radiolucent line on radiographs and mimic a fracture [4]. We report a case where the dilemma of an oblique radiolucent shadow on the femur, mimicking a distal extension of the proximal fracture, was encountered and addressed.

## Case presentation

**Figure 1:**
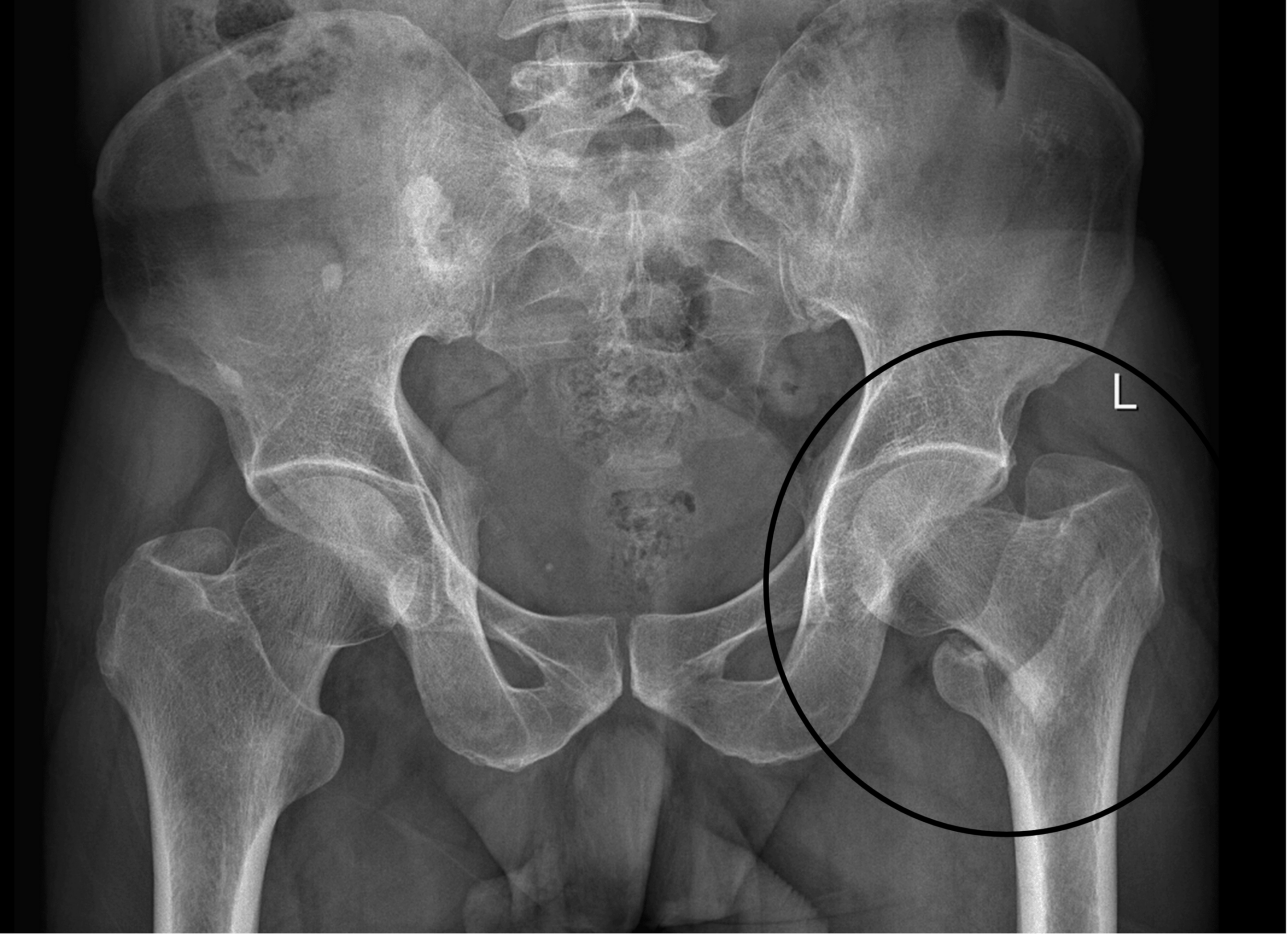
Plain radiograph of pelvis with bilateral hip showing left-sided AO 31A1.2 type of pertrochanteric femur fracture.

A 54-year-old male was admitted to our hospital following a fall on level ground. Radiographic examination revealed an AO 31A1.3 (AO/OTA 2018) type pertrochanteric fracture of the left femur (Figure 1) [5]. After ruling out other injuries, the patient was planned for closed reduction and internal fixation. As the patient had no comorbidities and the fracture exhibited a stable pattern, a decision was made to proceed with fixation using a short TFN (nail dimensions -10mm x 235mm, TFN-ADVANCED^TM ^Proximal Femoral Nailing System (TFNA) (DePuy Synthes, USA)).

**Figure 2:**
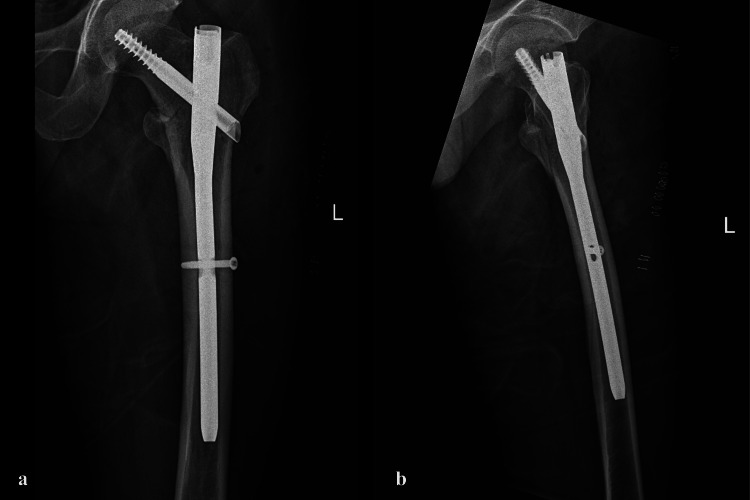
Post-operative (a) anteroposterior and (b) cross-table lateral radiographs showing stable internal fixation with a short TFN.

The patient was positioned supine on a fracture table, with the affected limb slightly internally rotated and the patella facing upwards. Closed reduction was achieved under fluoroscopic guidance. An incision was made proximal to the tip of the greater trochanter, and entry into the femoral canal was established with a guide pin. Proximal reaming was performed with a 16-mm reamer, and the guide wire was secured within the femoral canal. Sequential reaming was carried out in increments of 0.5 mm up to 11.5 mm and a 10mm x 235mm nail was selected. Following nail insertion, a lag screw was placed in the femoral head and neck proximally and distal locking screw was inserted. The procedure was uneventful (Figures 2a, 2b).

**Figure 3:**
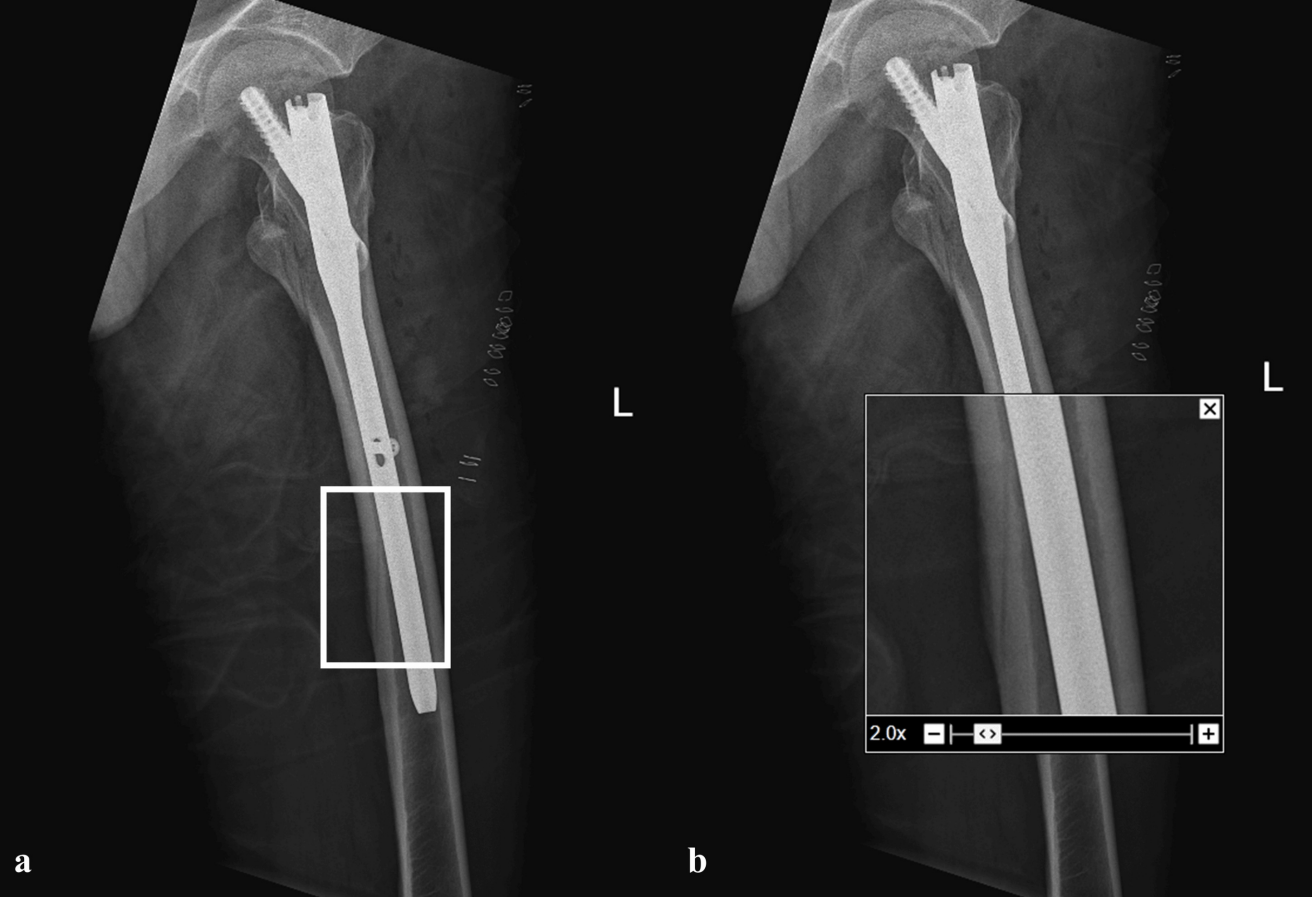
(a, b) Post-operative radiographs revealed presence of an oblique radiolucent line on the posterior cortex of femur adjacent to distal part of the nail in the middle third of femoral diaphysis.

**Figure 4:**
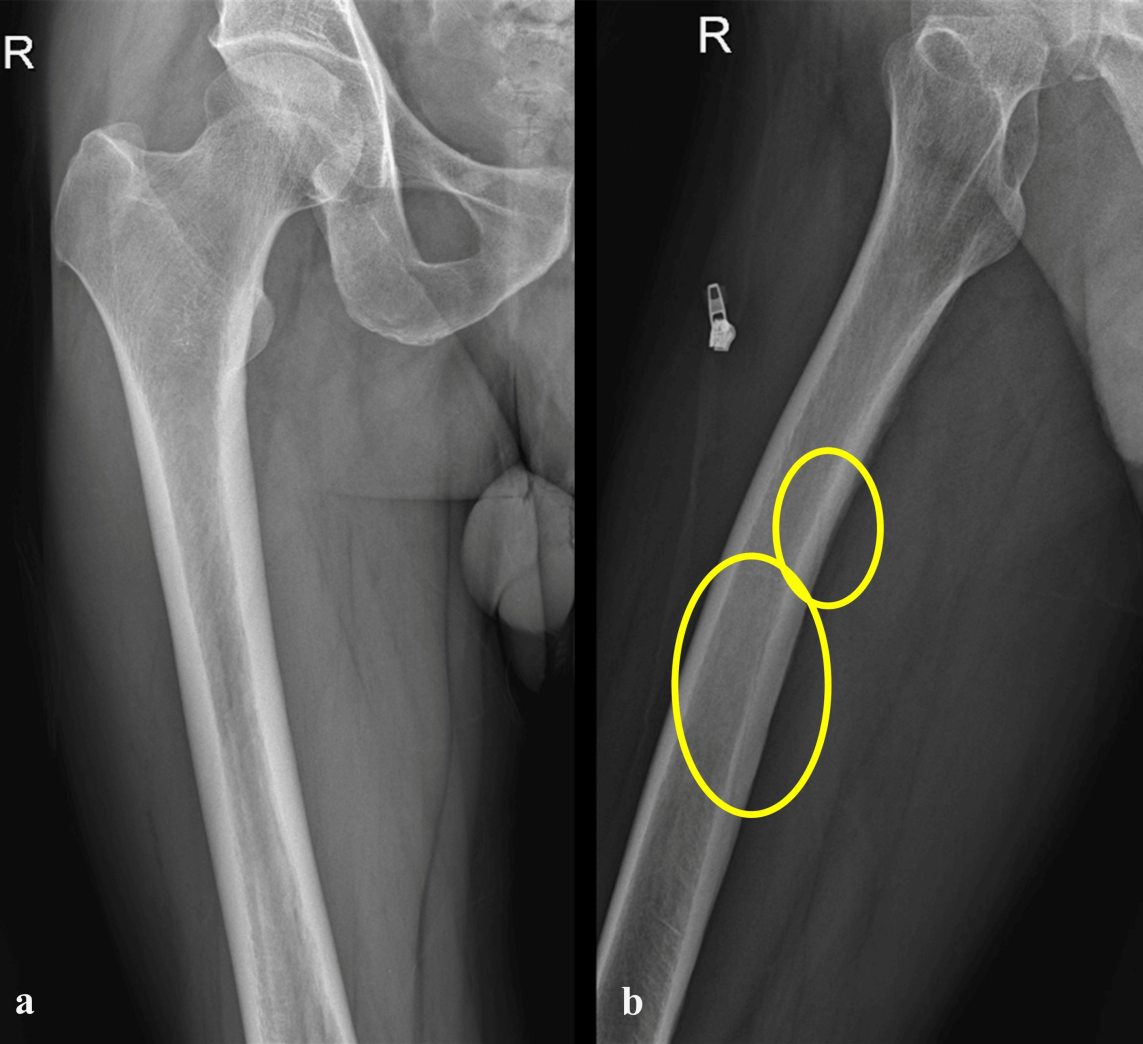
(a) Anteroposterior and (b) cross-table lateral radiographs of the contralateral side revealing two similar radiolucent lines on the posterior cortex of femur suggesting the shadow of the nutrient artery canals.

**Figure 5:**
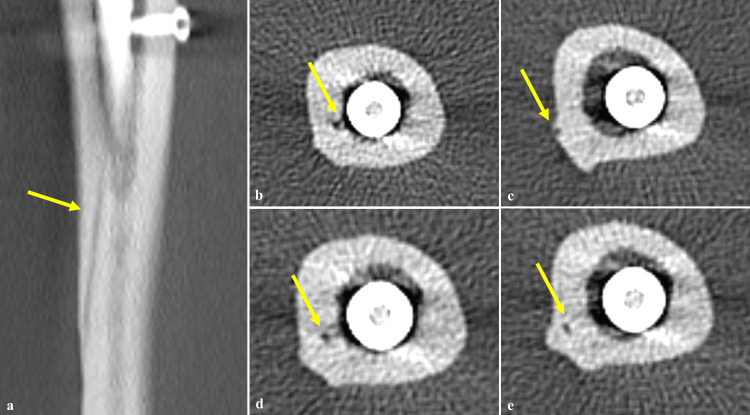
(a) Coronal and (b-e) axial CT images revealing features suggestive of nutrient artery canal (yellow arrows point at the nutrient artery canal).

However, post-operative radiographs revealed the presence of an oblique radiolucent line along the posterior cortex of the femur, adjacent to the distal part of the nail in the mid-diaphyseal region (Figures 3a, 3b). This raised suspicion of a possible undisplaced fracture. A full-length radiograph of the contralateral femur was obtained, which demonstrated a similar radiolucent line along the posterior cortex, suggestive of the nutrient artery canal (Figures 4a, 4b). To further clarify the finding, a CT scan of the femur was performed, which confirmed the presence of a nutrient artery canal without any evidence of a fracture line (Figures 5a-5e).

Clinically, the patient reported no pain in the mid-thigh region in the immediate post-op period, further supporting the conclusion that the radiolucent line was a normal anatomical variant rather than a fracture extension. Consequently, the patient was safely initiated on full weight-bearing mobilization without the need for revision to a longer nail.

## Discussion

Long bones receive their blood supply from a nutrient artery that enters obliquely through a nutrient foramen, typically directed away from the growing end of the bone [6]. In the femur and fibula, the nutrient foramen is usually located in the middle third, whereas in the tibia, it is typically found in the upper third [7].

Nutrient arteries are responsible for supplying approximately 70%-80% of the nutrients and oxygen required by long bones [7]. In the femur, the nutrient artery arises from the perforating branches of the deep femoral artery. After entering through the nutrient foramen, the artery traverses the cortex via the nutrient artery canal and extends into the medullary cavity [6,7]. On plain radiographs, nutrient artery canals can be seen as oblique or longitudinal radiolucent lines, resembling fracture lines. This can potentially lead the operating surgeon to overestimate the extent of the injury, influencing both the choice of implant and the post-operative mobilization strategy. For instance, the surgeon might be compelled to use a long TFN based on the false impression of a distal fracture. Similarly, detection of such a fracture mimic post-operatively could unnecessarily delay the initiation of full weight-bearing. Hence, a thorough knowledge and understanding of the radiographic appearance of nutrient artery canals and their differentiation from true fracture lines cannot be overstated.

The nutrient foramina of the femoral shaft are most commonly located on the medial lip of the linea aspera. Although in the majority of cases, nutrient foramina are observed in the middle third of the femoral shaft, some studies have reported their localization in the proximal one-third segment. Most studies describe the presence of at least one or two nutrient foramina in the femur [8]. The nutrient artery canal shadow may appear as an oblique cortical radiolucent line or as a longitudinal radiolucent line traversing the medullary cavity [9]. The visibility of the femoral nutrient canal in the cortex is very poor on anteroposterior (AP) radiographs but is much better appreciated on cross-table lateral (CTL) radiographs.

This discrepancy is attributed to several factors. First, the medullary cavity appears more radiolucent than the cortical bone on plain radiographs, potentially reducing the visibility of nutrient artery canals in both AP and CTL views. Additionally, the anatomical positioning of the nutrient artery canal, which typically enters the femur through the linea aspera in the postero-central region of the diaphysis, results in limited visibility on AP radiographs due to its obscuration by the anterior cortex. Furthermore, while the sclerotic margins of the nutrient artery canal enhance its radiographic visualization, these features are often obscured in AP views because of cortical bone overlap, whereas CTL views generally provide a clearer depiction.

Additionally, the orientation of the nutrient artery canal is typically upward and oblique in the cortical bone, a configuration that is more readily detected on CTL radiographs. Compared to true fracture lines, nutrient artery canals are typically seen involving only one part of the cortex, exhibit lower radiolucency, have a smaller diameter, and demonstrate blunt ends within both the cortex and medullary cavity. They often possess sclerotic margins within the cortex and follow a less linear path within the medullary cavity [9].

​Nutrient artery canals in the femur typically maintain a consistent appearance on postoperative radiographs, exhibiting unchanged characteristics over time. In contrast, fracture lines undergo a dynamic healing process, with their radiographic features evolving as healing progresses. Fracture lines become increasingly less well-defined on consecutive follow-ups until complete union is achieved [9].

## Conclusions

This case underscores the importance of recognizing the nutrient artery canal as a normal anatomical variant that may mimic a fracture line. Accurate identification using appropriate imaging can prevent misdiagnosis, avoid unnecessary changes in surgical planning, and allow timely post-operative mobilization. Importantly, awareness of this radiographic mimic is essential not only in proximal femoral fractures but in any type of femoral or long bone fracture, as misinterpretation can lead to overtreatment, including the use of unnecessarily long implants, more extensive dissection and surgeries, or unwarranted delay in rehabilitation. A thorough understanding of normal anatomical variants, such as the nutrient artery canal, is critical for optimizing patient outcomes across all fracture types.

In this case, the absence of pain in the mid-thigh region during the immediate post-op period further reinforced the benign nature of the radiolucent line and supported the decision to proceed with full weight-bearing mobilization. This aligns with and validates the radiological interpretation, emphasizing the value of correlating imaging findings with clinical assessment for sound decision-making.
